# Association of free-living diet composition with plasma lipoprotein(a) levels in healthy adults

**DOI:** 10.1186/s12944-023-01884-2

**Published:** 2023-09-05

**Authors:** Anastasiya Matveyenko, Heather Seid, Kyungyeon Kim, Rajasekhar Ramakrishnan, Tiffany Thomas, Nelsa Matienzo, Gissette Reyes-Soffer

**Affiliations:** 1 Department of Medicine, Columbia University Vagelos College of Physicians and Surgeons, New York, N.Y USA; 2 Irving Institute for Clinical and Translational Research, Columbia University, New York, N.Y USA; 3 Institute of Human Nutrition, Columbia University, New York, N.Y USA; 4 Center for Biomathematics, Department of Pediatrics, Columbia University Vagelos College of Physicians and Surgeons, New York, N.Y USA; 5 Department of Pathology and Cell Biology, Columbia University Vagelos College of Physicians and Surgeons, New York, N.Y USA

**Keywords:** Lipoprotein(a), Diet quality, Dietary components, Saturated fatty acids, HEI

## Abstract

**Background:**

Lipoprotein (a) [Lp(a)] is an apoB100-containing lipoprotein with high levels being positively associated with atherosclerotic cardiovascular disease. Lp(a) levels are genetically determined. However, previous studies report a negative association between Lp(a) and saturated fatty acid intake. Currently, apoB100 lowering therapies are used to lower Lp(a) levels, and apheresis therapy is FDA approved for patients with extreme elevations of Lp(a). The current study analyzed the association of free-living diet components with plasma Lp(a) levels.

**Methods:**

Dietary composition data was collected during screening visits for enrollment in previously completed lipid and lipoprotein metabolism studies at Columbia University Irving Medical Center via a standardized protocol by registered dietitians using 24 hour recalls. Data were analyzed with the Nutrition Data System for Research (Version 2018). Diet quality was calculated using the Healthy Eating Index (HEI) score. Fasting plasma Lp(a) levels were measured via an isoform-independent ELISA and apo(a) isoforms were measured using gel electrophoresis.

**Results:**

We enrolled 28 subjects [Black (*n* = 18); Hispanic (*n* = 7); White (*n* = 3)]. The mean age was 48.3 ± 12.5 years with 17 males. Median level of Lp(a) was 79.9 nmol/L (34.4–146.0) and it was negatively associated with absolute (grams/day) and relative (percent of total calories) intake of dietary saturated fatty acids (SFA) (R = -0.43, *P* = 0.02, SFA …(% CAL): R = -0.38, *P* = 0.04), palmitic acid intake (R = -0.38, *P* = 0.05), and stearic acid intake (R = -0.40, *P* = 0.03). Analyses of associations with HEI score when stratified based on Lp(a) levels > or ≤ 100 nmol/L revealed no significant associations with any of the constituent factors.

**Conclusions:**

Using 24 hour recall, we confirm previous findings that Lp(a) levels are negatively associated with dietary saturated fatty acid intake. Additionally, Lp(a) levels are not related to diet quality, as assessed by the HEI score. The mechanisms underlying the relationship of SFA with Lp(a) require further investigation.

**Supplementary Information:**

The online version contains supplementary material available at 10.1186/s12944-023-01884-2.

## Background

Atherosclerotic cardiovascular disease (ASCVD) is the leading cause of death in the United States [1]. One independent and causal risk factor for developing ASCVD is high plasma level of lipoprotein(a) [Lp(a)] [2–4]. Lp(a) has two main protein components: an integral membrane protein, apolipoprotein (apo) B100, covalently bound to the glycoprotein apolipoprotein(a) [apo(a)] [2–4]. Plasma Lp(a) levels are 70–90% determined by the *LPA* gene [5–7]. Apo(a) varies in size from 300 to 800 kDa due to different numbers of Kringle 4 type 2 (KIV-2) repeats, ranging from 1 to > 40. A universal consensus for the threshold of elevated Lp(a) associated with ASCVD risk has not been determined [8], hence there are multiple different published cut-off ranges. However, a continuous causal association between Lp(a) and ASCVD is well established [9].

Lifestyle modifications, including exercise and diet interventions, are low-cost and effective ways to prevent and help treat cardiovascular disease. Lp(a) levels do not change or may slightly increase (10–15%) after intense exercise training in previously sedentary individuals [10, 11]. Additionally, unlike other apoB-containing lipoproteins and CVD risk factors (i.e. obesity, insulin resistance), in which diet modifications contribute to a decreased risk of events [12], Lp(a) levels do not change during cardio-beneficial diet interventions [13, 14]. Several studies have examined the possible effects of dietary interventions on Lp(a) [15–18]. Studies by Ginsberg et al. [18], Shin et al. [15], and Silaste et al. [16] observed a negative relationship between plasma Lp(a) levels and saturated fatty acids (SFA) [15, 16, 18]. Conversely, Haring et al. found a positive relationship between plasma Lp(a) levels and unsaturated fatty acids [17]. The studies suggest that overall diet composition may influence Lp(a) levels and may not be in line with diets that provide cardiovascular benefits. To date, none of the studies directly evaluate the relationship between the participants' free-living diet and Lp(a) levels prior to intervention, which may or may not have contributed to the results on saturated fat and Lp(a) levels observed. Additionally, these studies did not include apo(a) isoform size and race/ethnicity, both known to affect Lp(a) levels [19]. Therefore, we examined food records from a diverse cohort of subjects previously enrolled for studies that evaluated lipid and lipoprotein metabolism. We evaluated the relationship of Lp(a) levels with diet composition, and diet quality as measured by the Healthy Eating Index (HEI) score.

## Methods

### Study participants

All studies were approved by the Columbia University Irving Medical Center (CUIMC) institutional review board (IRB), and informed consent was obtained from all participants. Participants were healthy volunteers with no history of cardiovascular disease (CVD) or type 2 diabetes (T2D) and did not report taking any lipid-lowering medications [20, 21]. Dietary data were obtained from screening visits for enrollment in previously completed lipid and lipoprotein metabolism studies at CUIMC. Only individuals with complete dietary records were included in the present analysis [20, 21].

### Study procedures

Participants were screened at our research center facilities after a 12 hour (hr) overnight fast. We recorded self-reported race/ethnicity (SRRE). Height and weight were measured using a scale, while wearing a hospital gown and no shoes. These measurements were used to calculate body mass index (BMI). Registered dietitians completed dietary 24 hr recalls, in person. Participants were excluded from this study if they followed non-conventional dietary habits such as the ketogenic diet or intermittent fasting. One dietary recall was obtained per participant via the multiple pass method [22, 23]. Dietary intake data were analyzed using Nutrition Data System for Research (NDSR) software Version 49 (2018) developed by the Nutrition Coordinating Center (NCC), University of Minnesota, Minneapolis, MN [24]. Diet macronutrient data were evaluated and included: carbohydrate, protein, and fat, including SFA, mono- (MUFA) and poly- (PUFA) unsaturated fatty acids as well as dietary fiber (soluble and insoluble). Diet composition was analyzed on an absolute [grams/day (g/d)] and relative basis [percent of total Calories (% Cal)]. This observational study examined the relationship of fasting Lp(a) levels with free-living diet composition, particularly fat intake.

### Healthy eating index

Microsoft Excel was used to calculate the Healthy Eating Index (HEI) score 2015 for each participant. HEI-2015 describes the diet quality according to the recommendations outlined in the 2015–2020 Dietary Guidelines for Americans by generating a score from 0 to 100 (100 being 100% in congruence with the guidelines) [25]. The score is a composite of thirteen factors representing different food groups classically associated (positively or negatively) with chronic disease. The relationship between HEI score and Lp(a) level was evaluated in all subjects and in subgroups stratified by Lp(a) levels. There is no clinically accepted level to denote high Lp(a), therefore, we stratified our cohort into "high" and "low" using a cut point that considers two published recommendations for Lp(a) levels (“high”—> 100 nmol/L; “low”—≤ 100 nmol/L). The National Heart, Lung, and Blood Institute (NHLBI) Working Group Recommendations use Lp(a) levels > 75 nmol/L as "high" [26], and the 2019 European Society of Cardiology (ESC)/European Atherosclerosis Society (EAS) Guidelines consider elevated Lp(a) levels as Lp(a) ≥ 125 nmol/L [27].

### Laboratory measurements

Each participant had a 12-hr fasting blood draw via an intravenous (IV) catheter from forearm veins. Briefly, blood was obtained in EDTA containing test tubes, immediately placed on ice, and spun in a centrifuge at 1693 rotational centrifugal force, 4° Celsius (C) for 20 min. Plasma was isolated from the test tube and stored in a -80° C freezer. Frozen samples were shipped on dry ice to the laboratory of Dr. Santica Marcovina (Seattle, Washington), where plasma Lp(a) levels were measured using an isoform-independent, double monoclonal antibody-based enzyme-linked immunosorbent assay (ELISA) [28–30]. Lp(a) levels were not normally distributed, so we calculated medians and interquartile range (IQR). Apo(a) isoform size measurements were performed by the same laboratory [31], which is currently the best available method as not all genetically determined isoforms of apo(a) are expressed and can be found in circulation as an Lp(a) particle. Plasma lipids (total cholesterol (TC), triglycerides (TG), and high-density lipoprotein (HDL) cholesterol) were measured by Integra400plus (Roche). Plasma low-density lipoprotein (LDL) cholesterol (C) levels were estimated using the Friedewald formula. Plasma apoB100 was measured using ELISA kit # 3715-1HP-2 from Mabtech, Inc, Cincinnati, OH.

### Weighted Isoform Size (wIS) calculations

Most individuals express two apo(a) isoforms in plasma that vary in size, the smaller isoforms are dominant and inversely correlated with Lp(a) plasma levels. To account for the difference in percent expression (determined by gel electrophoresis) of each isoform, we calculated a weighted isoform size [32]. Example: If the two allele sizes are 20 and 30, with relative expression of 70% and 30%, respectively, the *wIS* is 0.7*20 + 0.3*30 = 23.

### Statistical analysis

Based on previously identified relationships of diet components with lipids, twenty-three dietary variables were identified a priori for analysis from the 170 variables available via NDSR output. Diet data are presented as absolute (g/d) and relative (% Cal) intake. Pearson correlation and linear regression were used to evaluate relationships between variables using the R software [33]. Apo(a) isoform size and SRRE are determinants of plasma Lp(a) levels [19], as such we control for these variables in our linear regression models. Unpaired t-test was used to analyze HEI score differences by stratifying subjects into two Lp(a) groups (≤ 100 nmol/L and > 100 nmol/L). Statistical significance was set at a *P*-value less than or equal to 0.05.

## Results

Twenty-eight participants met the inclusion criteria for the study. Baseline characteristics including lipid and lipoprotein levels are listed in Table 1. The mean age of the cohort was 48.3 ± 12.5 years; 17 out of the 28 subjects were male and 18 listed Black as their SRRE. The participants were overweight with a mean BMI of 29.5 ± 3.3 kg/m^2^. Plasma lipid levels (TC, TG, HDL-C, LDL-C) and apoB100 levels were within normal ranges. The median Lp(a) level was 79.9 nmol/L (IQR 34.4—146 nmol/L) and the calculated *wIS* was 22.4. As observed in larger published cohorts, apo(a) isoform size was negatively associated with Lp(a) levels (Supplemental Fig. 1). Individual Lp(a) levels, isoform size expressed, and calculated *wIS* for the full cohort are presented in Supplemental Table 1.

**Table 1:** Participant characteristics (*n* = 28)

**Sex** n(%)	
* Male*	17 (60.7)
**Race/Ethnicity** n(%)	
* Black*	18 (64.3)
* Hispanic*	7 (25.0)
* White*	3 (10.7)
**Age** (years)	48.3 ± 12.5
**BMI** (kg/m^2^)	29.5 ± 3.3
**Total Cholesterol** (mg/dL)	152.8 ± 22.3
**Total Triglyceride** (mg/dL)	98.5 ± 43.6
**LDL-C** (mg/dL)	86.6 ± 18
**HDL-C** (mg/dL)	46.6 ± 12.8
**ApoB100** (mg/dL)	90.7 ± 27.2
**Lp(a)** (nmol/L)	79.9 (34.4–146.0)
**Apo(a) *wIS***	22.4 ± 4.6
**HEI score**	57.1 ± 16

Data are presented as mean ± SD, median (IQR), or n (%)

*BMI* Body Mass Index, *LDL-C* Low-Density Lipoprotein Cholesterol, *HDL-C* High-Density Lipoprotein Cholesterol, *ApoB100* Apoprotein (B100), *Lp(a)* Lipoprotein (a), *Apo(a)* Apoprotein (a), *HEI* Healthy Eating Index, *n* number, *kg* kilogram, *m2* meter squared, *mg* milligrams, *dL* deciliter, *IQR* Interquartile Range, *SD* Standard Deviation

**Fig. 1:**
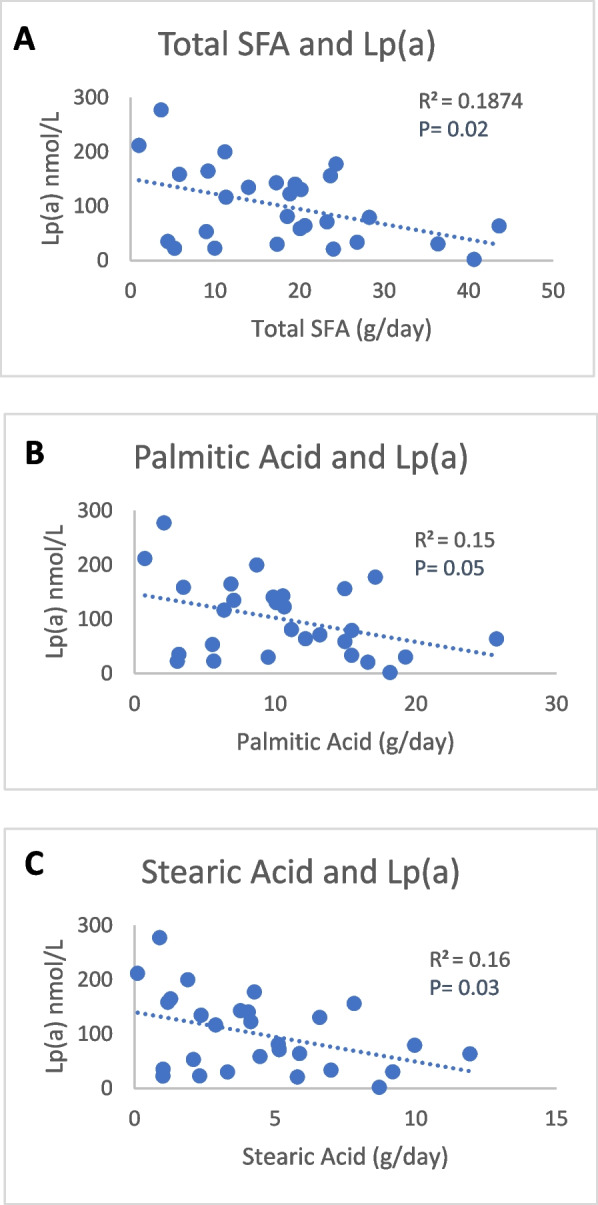
Title: Relationship between Saturated Fatty Acids, Palmitic Acid, Stearic Acid and Lp(a). Legend: Scatter plot of percent calories from SFA (**A**), Palmitic acid (**B**) and Stearic Acid (**C**) with Lp(a) with a best fitted line. Pearson correlation was done to obtain the p-value. SFA, Saturated Fatty Acid; Lp(a), lipoprotein(a) Alpha significance set at *p* ≤ 0.05

### Relationships between dietary components—plasma lipids, lipoproteins and Lp(a)

As expected, apoB100 levels were positively and significantly correlated with absolute and relative values of total fat and particularly absolute and relative intake of SFA. (Table 2).

**Table 2:** Relationship between Apo lipoprotein B100 and dietary factors

	Absolute		Relative	
	R	*P*-value	R	*P*-value
Total Fat	0.40	0.036^*^	0.52	0.005^*^
SFA	0.58	0.001^*^	0.62	< 0.001^*^

*SFA* Saturated fatty acid

^*^- significant *P*-value of ≤ 0.05

### Relationship of Lp(a) with macronutrients

The mean intakes of absolute and relative total carbohydrate, protein, and fat in our participants can be seen in Table 3. We found no relationship between average energy intake (kcal/d) and Lp(a) concentration (R = -0.32, *P* = 0.10). A moderate negative relationship was observed between fat intake (g/d) and Lp(a) levels (*P* = 0.08), but this relationship did not persist when normalized to percent of total calories (*P* = 0.13). We observed no relationships between plasma Lp(a) levels and carbohydrate or protein intake (Table 3). However, when we included *wIS* and SRRE in our model we observed a positive trend between Lp(a) and relative intake of carbohydrates (*P* = 0.07) and a negative relationship between fat (*P* = 0.05) and Lp(a) levels (Table 3).

**Table 3:** Relationship between dietary variables and Lp(a)

**Dietary Variables**		**Absolute Intake**			**Relative Intake**			**Absolute Intake**^a^		**Relative Intake**^a^	
		Mean ± SD	R	*P*-value	Mean ± SD	R	*P*-value	R^2^	*P*-value	R^2^	*P*-value
		g/d			% Cal						
**Total Energy**	**kcal/d**	1722.6 ± 599.8	-0.32	0.1	NA	NA	NA	0.46	0.203	NA	NA
**Macronutrients**	Carbohydrate	221.8 ± 87.7	-0.18	0.35	50.7 ± 12.5	0.24	0.22	0.42	0.617	0.5	0.07
	Protein	79.5 ± 35.9	-0.27	0.17	18.5 ± 5.8	0.03	0.90	0.47	0.156	0.42	0.66
	Fat	60.5 ± 29.9	-0.34	0.08	30.5 ± 11.7	-0.29	0.13	0.48	0.108	0.51	0.05
**Saturated Fatty Acids**	Total SFA	18.1 ± 10.8	-0.43	0.02^*^	9.2 ± 4.9	-0.38	0.04^*^	0.52	0.04^*^	0.53	0.03^*^
	Palmitic Acid	10.6 ± 6	-0.38	0.05^*^	7.7 ± 4.1	-0.33	0.08	0.52	0.047^*^	0.53	0.03^*^
	Stearic Acid	4.4 ± 3.1	-0.4	0.03^*^	2.3 ± 1.4	-0.37	0.06	0.52	0.036^*^	0.54	0.02^*^
**Unsaturated Fatty Acids**	Total MUFA	22.8 ± 13.1	-0.14	0.47	11.4 ± 5.5	-0.09	0.66	0.43	0.529	0.43	0.54
	Palmitoleic Acid	1.2 ± 0.9	-0.26	0.18	0.6 ± 0.5	-0.17	0.4	0.44	0.308	0.43	0.44
	Oleic Acid	20.9 ± 12.2	-0.13	0.51	10.6 ± 5.1	-0.08	0.7	0.43	0.55	0.43	0.56
	Total PUFA	14.1 ± 8.9	-0.26	0.18	7.1 ± 3.3	-0.18	0.37	0.47	0.152	0.47	0.14
	Linoleic Acid	12.0 ± 8.2	-0.25	0.20	6.1 ± 3.1	-0.18	0.37	0.47	0.149	0.47	0.13
	Linolenic Acid	1.4 ± 0.7	-0.37	0.05	0.71 ± 0.3	-0.25	0.19	0.49	0.092	0.46	0.19
**Dietary Fiber**^b^	Soluble Fiber	6.3 ± 4.2	-0.08	0.67	3.69 ± 1.85	0.1	0.6	0.43	0.466	0.42	0.91
	Insoluble Fiber	15.4 ± 11.3	-0.09	0.64	8.95 ± 6.31	0.02	0.92	0.43	0.48	0.46	0.19

*SD* Standard deviation. Pearson correlation was used to evaluate the relationships between variables. *d* day, *g* grams, *Kcal* kilocalories, *NA* Not Applicable

^a^Linear regression was used to evaluate the relationships between variables, while controlling for wIS and SRRE. wIS, weighted Isoform Size; SRRE, Self-Reported Race/Ethnicity

^*^- significant P-value of ≤ 0.05

^b^—Relative intake values are calculated as % calories using g/1000 cal

### Effects of saturated fatty acids—palmitic acid and stearic acid on Lp(a)

The mean intakes of absolute and relative amounts of SFA, palmitic acid, and stearic acid intake are reported in Table 3. There was an inverse relationship between Lp(a) levels with dietary SFA [absolute (R = -0.43, *P* = 0.02) and relative (R = -0.38, *P* = 0.04) (Fig. 1A)], dietary palmitic acid [absolute (R = -0.38, *P* = 0.05) (Fig. 1B)], and dietary stearic acid [absolute (R = -0.40, *P* = 0.03) (Fig. 1C)]. These relationships persisted when controlling for *wIS* and SRRE. Extrapolation of these findings suggest that for every one percent increase in calories from SFA, Lp(a) levels decrease by 5.97 nmol/L. We also observed trends toward a negative correlation between Lp(a) and relative intake of palmitic (R = -0.33, P = 0.08) and stearic acid (R = -0.37, P = 0.06).

### Effects of unsaturated fatty acids—monounsaturated fatty acids and polyunsaturated fatty acids on Lp(a) levels

The mean absolute and relative values of total MUFA, palmitoleic acid, and oleic acid intake in our population are listed in Table 3. The average daily PUFA, linoleic acid, and linolenic acid intake are also reported in Table 3. Plasma Lp(a) levels were not associated with absolute (g/d) or relative (% calories) MUFA or PUFA intake, even when adjusted for *wIS* and SRRE.

### Effects of dietary fiber on Lp(a) levels

The mean soluble and insoluble fiber intake values are reported in Table 3, and there were no significant relationships with Lp(a) even after adjusting for *wIS* and SRRE.

### Relationships of Lp(a) with healthy eating index score

**Fig. 2:**
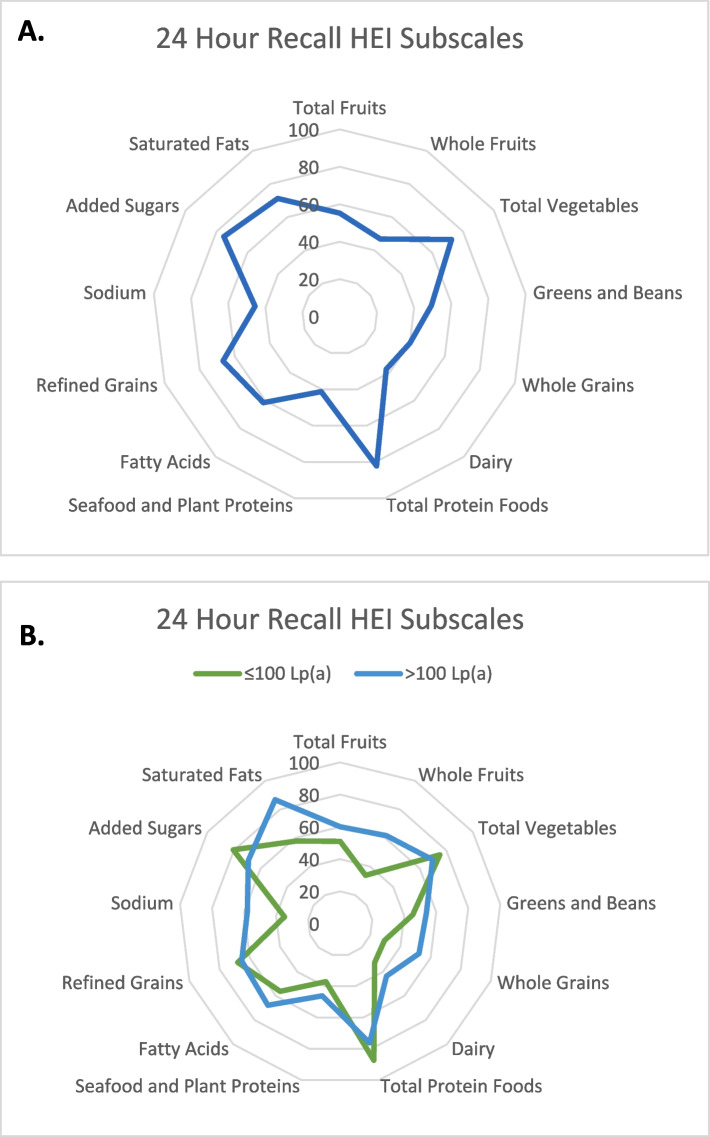
Radar Plots for Healthy Eating Index Broken Down by Food Groups **A**. Overall HEI assessment based on individual food groups that make up HEI score. **B**. Unpaired t-test, presenting the relationships between high and low Lp(a) levels with whole fruits (P = 0.10), sodium (*P* = 0.14), whole grains (*P* = 0.15), saturated fats (*P* = 0.03). HEI, Healthy Eating Index; Lp(a), Lipoprotein(a) Alpha significance set at *P* < 0.05

**Fig. 3:**
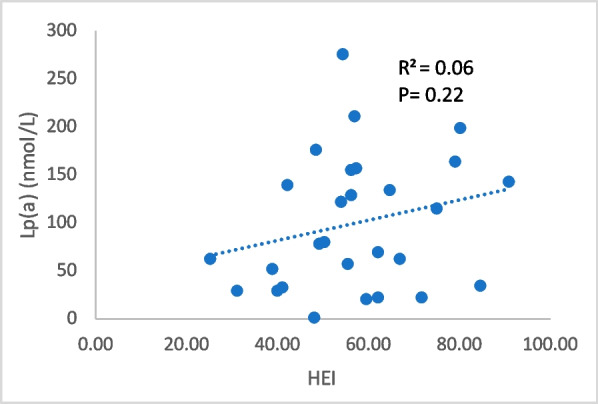
Relationship between Lp(a) and HEI. Lp(a): Lipoprotein(a); HEI: Healthy Eating Index

Our cohort had an average HEI score of 57.1 ± 16, this is similar to results published by National Health and Nutrition Examination Survey (NHANES), which found the average HEI score for Americans is 58 [34], suggesting that our sample reflects dietary patterns previously described throughout the USA population. The score calculation is made of 13 food components, which we graphically represent for our cohort in Fig. 2A. An HEI score of 100 would suggest perfect alignment with the dietary guidelines. We investigated the relationship between our cohort's HEI index score and Lp(a) levels (Fig. 3).

There was no statistically significant relationship between HEI score and Lp(a) level (Fig. 3) even after adjusting for *wIS* and SRRE (*P* = 0.13). We examined whether this relationship varied in individuals with high versus low Lp(a) level but found no differences between the groups (*P* = 0.09) (Table 4). Further, when analyzed for each of the thirteen dietary subgroups that make up the HEI score by unpaired t-test between low and high Lp(a) (Fig. 2B), only dietary saturated fat reached statistical significance (*P* = 0.03).

**Table 4:** Relationship between HEI score and Lp(a) value stratified by normal and high Lp(a) levels (*N* = 28)

	**HEI Score** Mean ± SD	*P*-value
**Lp(a) Value-Low** ≤ 100 nmol/L (*n* = 15)	52.4 ± 16	0.09
**Lp(a) Value-High** > 100 nmol/L (*n* = 13)	62.6 ± 14.2	

Unpaired t-test was used to evaluate the relationships between variables

*HEI* Healthy Eating Index, *SD* Standard Deviation

Alpha significance set at P ≤ 0.05

## Discussion

The current study aimed to evaluate the relationships between a free-living diet and Lp(a) plasma levels. These relationships had been studied in controlled settings (randomized studies), yet we wanted to see if the previous reports were also found in free-living environments. Our study population was small, however the small group showed similar positive relationships between plasma apoB100 levels and TC (R = 0.41, *P* = 0.03), and LDL-C (R = 0.52, *P* = 0.005) which have been found in larger cohorts, validating the relationship of apoB100 with plasma lipids. Moreover, they showed positive relationships between plasma apoB100 with dietary total fat and SFA, validating the previously relationships between diet and lipoproteins in this small cohort [35].

Plasma Lp(a) levels are strongly determined by genetics [5–7], and high levels of Lp(a) are a causal risk factor for ASCVD [31]. Heart healthy diets and lifestyle changes are the first steps to decreasing cardiovascular risk [14], yet these have not been shown to lower Lp(a) levels.

Previous results from the OMNI study showed a significant increase in Lp(a) levels with different macronutrient rich controlled diets [change in Lp(a) mean from baseline (*P*-value): carbohydrate + 3.2 (< 0.001); protein + 4.7 (< 0.001); unsaturated fat + 2.1(< 0.001)] [17]. However, in our data set controlling for *wIS* and race, we find no correlations between Lp(a) levels and protein or unsaturated fat, and a positive trend with relative intake of carbohydrates.

Current Dietary Guidelines for Americans (2020–2025) recommend consuming less than 10% of daily calories from saturated fat [36]. The current study supports previous findings from controlled diet studies that show high SFA content associates with lower levels of Lp(a) [15, 16, 18]. Using the average and standard deviation of SFA intake observed in our cohort (9.2 ± 4.9% Cal) and our linear regression model which includes *wIS* and SRRE, we estimate that an individual with an Lp(a) level of 100 nmol/L in whom SFA intake increases, for example, from 4 to 9%, will experience a decrease in their Lp(a) level to 70.13 nmol/L.

Our data on free-living diets can be compared to controlled studies such as the Delta study. In that study, three different controlled diet compositions were investigated [average American diet (AAD) (37% Fat, 16% SFA, 14% MUFA, 7% PUFA); step-1 diet (30% Fat, 9% SFA, 14% MUFA, 7% PUFA); low saturated fat diet (Low Sat) (26% Fat, 5% SFA, 14% MUFA, 7% PUFA)] and Lp(a) levels decreased as the percent of SFA of total calories was increased [AAD: (%kcal SFA = 15.0 ± 0.4, Lp(a) = 15.5 ± 1.8 mg/dL); step-1: (%kcal SFA = 9.0 ± 0.1, Lp(a) = 17.0 ± 1.8 mg/dL); low sat: (%kcal SFA = 6.1 ± 0.5, Lp(a) = 18.2 ± 1.9 mg/dL)] [18]. Two additional studies, one by Shin et al. observed Lp(a) increases similar to those observed in Delta study as participants switched from a high fat/low carb diet (40% Fat, 13% SFA, 11% MUFA, 13.8% PUFA, 3.4% trans-fat, 45% carbohydrate, 15% protein) to a low fat/high carb diet (20% Fat, 4.9% SFA, 9.9% MUFA, 5.1% PUFA, 2.4% trans-fat, 65% carbohydrate, 15% protein), from an Lp(a) level of 8.91 (IQR 3.41 – 34.6) mg/dL to 11.47 (IQR 3.84 – 38.78) mg/L, respectively. Silaste et al. examined how dietary fat and vegetable consumption affected lipid levels. Using baseline measurements and two separate diets [baseline (36 ± 6 percent of total energy intake (E%) from fat, 15 ± 3 E% SFA, 14 ± 3 E% MUFA, 6 ± 1 E% PUFA, 46 ± 7 E% carbohydrate, 17 ± 2 E% protein), low fat with low vegetable (LFLV) consumption (31 E% fat, 11 E% SFA, 13 E% MUFA, 7 E% PUFA, 49 E% carbohydrate, 20 E% protein) and low fat with high vegetable (LFHV) consumption (31 E% fat, 9.5 E% SFA, 11 E% MUFA, 9.5 E% PUFA, 50 E% carbohydrate, 20 E% protein)], the authors found that Lp(a) levels increased by 7% from baseline to LFLV diet and 9% from baseline to LFHV diet. More recently, a study by Ebbeling et al., showed that Lp(a) levels went down significantly (14.7%) when subjects consumed a low carbohydrate, high fat diet (60% of total energy from fat, 21% SFA, 25% MUFA, 11% PUFA, 20% carbohydrate, 20% protein) [37] compared to moderate-carbohydrate diet (40% of total energy from fat, 14% SFA, 16% MUFA, 9% PUFA, 40% carbohydrate, 20% protein) and high-carbohydrate diet (20% of total energy from fat, 7% SFA, 8% MUFA, 5% PUFA, 60% carbohydrate, 20% protein), where Lp(a) decreased by 2.1% and increased by 0.2% without significance, respectively. A similar observation was recently described in the GET-READI, randomized crossover feeding study. In this study conducted in African American population, participants either consumed the American diet with 16% SFA or dietary approaches to stop hypertension (DASH) diet with 6% SFA, for 5 weeks. Lp(a) levels were 44 mg/dl on the 16% SFA diet and 58 mg/dL with the 6% SFA diet [38]. However, in another study, where participants consumed frozen plant-based meals for 5 weeks during Lent (SFA 4.7% Kcal), researchers observed a significant reduction in Lp(a) by 10% (from 56 to 51 mg/dL) [39]. The differences in the findings reported in the latter study could be due to presence of hypertension and diabetes in the study subjects as the previous studies reported findings in otherwise healthy populations.

Our findings show a higher effect size (by a factor of 6 to 8) compared to the Delta study and the other two crossover studies (Shin, Silaste) (Supplemental Fig. 2). One reason can be our small cohort and the cross-sectional nature of the study. However, our ability to replicate effects of SFA on Lp(a) in free-living environments highlights its role in regulating Lp(a) levels. However, the possibility of one or two outliers influencing the regression coefficient in our small cohort fitted with two continuous variables (*wIS*, SFA%) and three SRRE categories is real. When we looked further into intercorrelations among the predictor variables that we examined, we found that SFA% was negatively correlated with *wIS* and was higher in Blacks compared to the other two SRRE groups. With our current knowledge, these relationships have no biological basis and could have arisen by chance. Since the SFA% was significantly correlated with Lp(a) level only in the presence of *wIS* and SRRE and  both correlated separately with SFA%, we would like to be conservative and conclude that the effect of SFA% on Lp(a) level is negative, while the magnitude of the effect may be overestimated due to the small cohort. The three crossover studies (Delta, Shin, Silaste) do not have this concern since each subject was studied at different SFA% levels, and so each subject served as their own control.

The current study adds another body of evidence, including apo(a) isoform size and SRRE, that support higher SFA diets associated with low Lp(a) levels. Neither our study nor previous studies have performed metabolic studies that could help elucidate the mechanisms that are regulating these reported associations. We hypothesize that the lower Lp(a) levels with diets high in SFA could be due to decreased production of Lp(a) particles.

A mechanism for this relationship could be that SFA intake changes the fatty acid profile in the phospholipid membrane of an apoB100-containing particles in the liver (main protein that binds to apo(a) in the liver) and thereby regulates the synthesis and secretion of Lp(a) particles. There are no data reported for understanding the effects of macronutrients on synthesis or production of Lp(a) particles.

Another mechanism proposed by Enkhmaa et al., suggest that lower SFA diets could reduce the clearance of Lp(a) particles via the LDL receptor (LDLr) [40]. This could be attributable to increased competition with other apoB100 containing particles, including LDL. SFA have been shown to interfere with the formation of cholesterol esters and through accumulation of cholesterol preventing activation of the sterol receptor binding protein which can downregulate LDLr which has been proposed as one of the mechanisms whereby Lp(a) particles can get cleared [41]. A recent study from our group showed that both production and clearance of Lp(a) and isoforms regulate its level [32]. Therefore, we speculate that diet composition may be regulating Lp(a) levels through combined mechanisms.

## Study strengths and limitations

Although a small study, our calculated diet quality based on HEI analysis is similar to those reported in larger cohort studies [34]. The HEI score provides a way to assess diet quality, and we hypothesized that a higher HEI score (better overall diet quality) would correlate with a lower level of Lp(a). However, we found no significant relationship between HEI and Lp(a) levels including saturated fat. Importantly, when stratified by Lp (a)level (≤ or > 100 nmol/L), we observed a statistically significant relationship with saturated fat that makes up the HEI score. The low Lp(a) group had a lower score for saturated fat (58%) [compared to high (87%)], meaning they consumed more saturated fat and thus received a lower score when HEI was calculated [25]. This data supports our overall findings on the negative relationship of SFA with Lp(a) levels.

Our study has several limitations: (1) we had a small sample size based on availability of complete food records and low enrollment numbers that are needed to complete our metabolic studies, and (2) the study was observational in nature. There are known limitations to 24-hr dietary intake data such as recall bias, which may be skewed based on the subject’s desire to express their intake to the recorder. It is possible that participants under or over-reported various foods or left out stereotypically undesirable foods entirely. Photographic or meal-logging systems could have been used to help minimize this bias, however these dietary assessment tools may impart additional bias as participants may consciously or subconsciously change their intake whenever diet information is collected. Additionally, only one recall per participant was analyzed for this study. Despite these limitations, the study population did have similar SFA intake compared to larger controlled randomized diet studies that have similar findings.

## Conclusion

Our findings support that increased dietary saturated fat is associated with low Lp(a) levels. However, the mechanisms regulating these relationships need to further be investigated as diets high in SFA have been linked to adverse cardiovascular risk. Together with the growing field of nutrigenomics [42], it is possible that individualized diet recommendations can be tailored to address a patient's ASCVD risk profile, and determine what is best for individuals with high levels of Lp(a).

### Supplementary Information

**Additional file 1.**

## Data Availability

The datasets generated during and/or analyzed during the current study are available in the LabArchives upon request.

## Abbreviations

ASCVD: Atherosclerotic cardiovascular disease
[Lp(a)]: Lipoprotein(a)
apo: Apolipoprotein
[apo(a)]: Apolipoprotein(a)
KIV-2: Kringle 4 type 2
SFA: Saturated fatty acids
HEI: Healthy Eating Index
IRB: Institutional review board
CVD: Cardiovascular disease
T2D: Type 2 diabetes
CUIMC: Columbia University Irving Medical Center
SRRE: Self-reported race/ethnicity
BMI: Body mass index
NDSR: Nutrition Data System for Research
NCC: Nutrition Coordinating Center
MUFAs and PUFAs: Mono-and poly- unsaturated fatty acids
NHLBI: National Heart, Lung, and Blood Institute
ESC: European Society of Cardiology
EAS: European Atherosclerosis Society
IV: Intravenous
IQR: Interquartile ranges
TC: Total cholesterol
TG: Triglycerides
HDL: High-density lipoprotein
LDL: Low-density lipoprotein
ELISA: Enzyme-linked immunosorbent assay
wIS: Weighted isoform size
LFLV: Low fat with low vegetable
LFHV: Low fat with high vegetable
DASH: Dietary Approaches to Stop Hypertension
NHANES: National Health and Nutrition Examination Survey
NCEP ATP: The National Cholesterol Education Program Adult Treatment Panel
AHA: American Heart Association
TLC: Therapeutic lifestyle changes
