# Supplementary material for: Association of free-living diet composition with plasma lipoprotein(a) levels in healthy adults
Source: Lipids Health Dis. 2023 Sep 5;22:144. doi: 10.1186/s12944-023-01884-2 (PMC10478368; doi:10.1186/s12944-023-01884-2)
Supplement: Supplementary file 1 — Additional file 1. [file 12944_2023_1884_MOESM1_ESM.pdf]

Supplemental Data

Supplemental Table 1. Individual Subject Data

Subject Number	Lp(a) (nmol/L)	Small Isoform	% Smaller	Large Isoform	% Larger	wIS	SRRE
1	30.1	31	86	34	14	31.42	B
2	158.3	18	61	20	39	18.78	B
3	140.3	16	82	22	18	17.08	B
4	142.7	22	79	26	21	22.84	B
5	70.9	21	97	30	3	21.27	B
6	1.6	18	7	35	93	33.81	B
7	63.8	18	50	19	50	18.5	B
8	33.2	22	6	26	94	25.76	B
9	130.3	22	92	26	8	22.32	B
10	80.8	22	79	25	21	22.63	B
11	155.8	15	100	[redacted]		15	W
12	134.4	19	100	[redacted]		19	H
13	211.5	17	87	30	13	18.69	H
14	164.4	26	55	29	45	27.35	B
15	177.2	17	72	23	28	18.68	B
16	22.2	26	100	[redacted]		26	W
17	20.6	17	12	24	88	23.16	H
18	29.7	23	60	34	40	27.4	H
19	116.4	17	100	[redacted]		17	H
20	199.7	18	100	[redacted]		18	H
21	277	15	44	21	56	18.36	B
22	22.5	14	61	28	39	19.46	H
23	34.8	28	60	30	40	28.8	W
24	79	25	89	27	11	25.22	B
25	122.4	23	100	[redacted]		23	B
26	63.3	19	59	27	41	22.28	B
27	58.4	24	100	[redacted]		24	B
28	52.9	22	100	[redacted]		22	B

Legend: Lp(a)wIS – weighted Isoform Size; SRRE – Self Reported Race/Ethnicity. Large isoform ≥22, Small isoform<22.

Supplemental Table 2. Relationship Between ApoB100 and Dietary factors

	Absolute		Relative	
	R	p-value	R	p-value
Total Fat	0.40	0.036	0.52	0.005
SFA	0.58	0.001	0.62	<0.001

*SFA: Saturated fatty acid

Supplemental Figure 1. Negative Relationship between Lp(a) and *wIS*

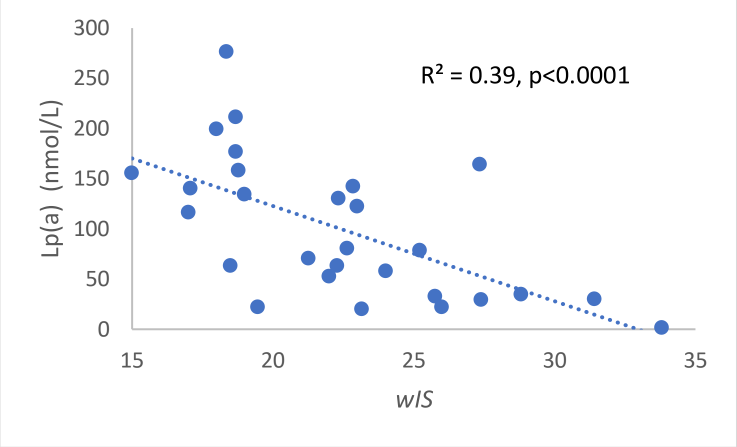

Lp(a): Lipoprotein(a); *wIS*: weighted Isoform Size

Supplemental Figure 2. No Relationship between Lp(a) and HEI

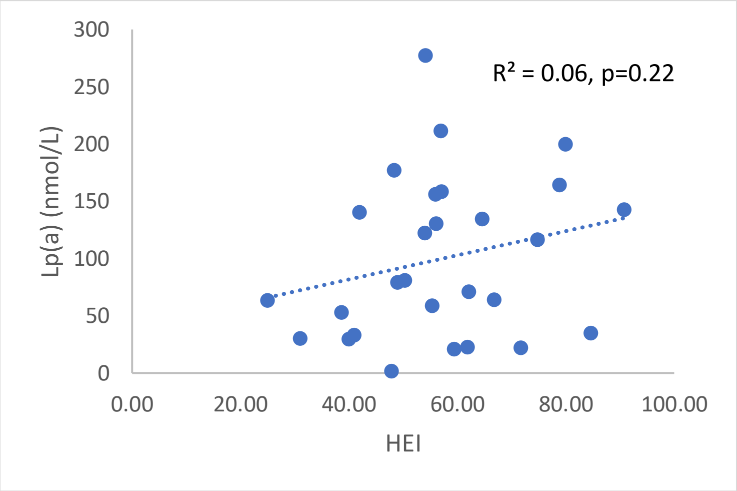

Lp(a): Lipoprotein(a); HEI: Healthy Eating Index

Supplemental Figure 3. Relationship between Lp(a) and Percent Calories from SFA in two different studies.

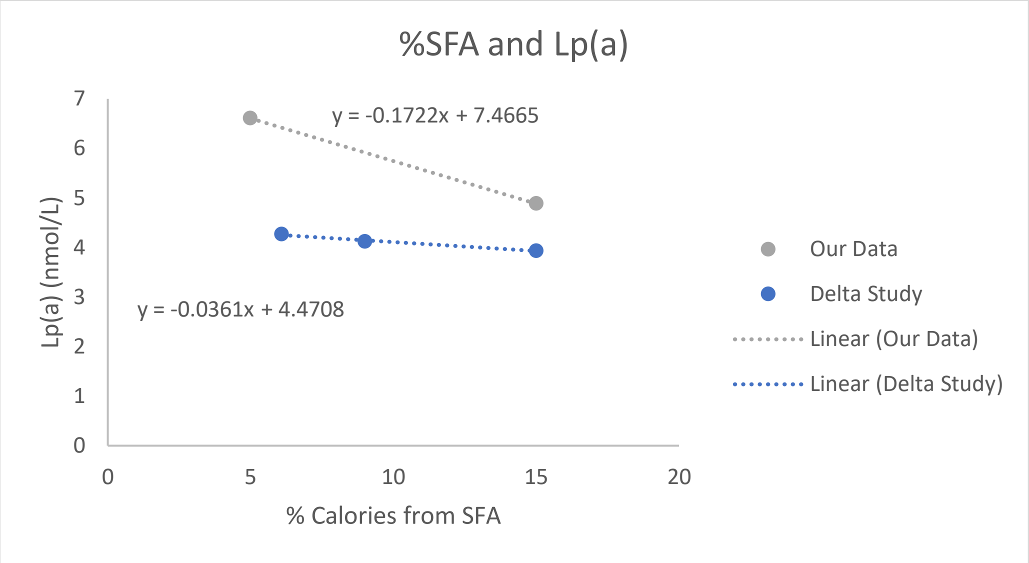

Delta Study: line plotted from reported Lp(a) values (in mg/dL and square rooted) and percent calories from SFA in the published Delta study.

Our Data: line plotted from the slope of a scatter plot from our Lp(a) values (nmol/L converted to mg/dL; square rooted to match the Delta study) and percent calories from SFA we report in this paper.
